# Dental, mandibular and parotid gland radiation doses in curatively treated head and neck squamous cell carcinoma: a retrospective cohort study

**DOI:** 10.1038/s41405-026-00416-1

**Published:** 2026-04-02

**Authors:** Veera Emilia Kärkkäinen, Henna Hietala, Petri Koivunen, Juha Nikkinen, Kaisa Lehtiö

**Affiliations:** 1 Department of Oncology and Radiotherapy, Oulu University Hospital, Oulu, Finland; 2 Department of Otorhinolaryngology-Head and Neck Surgery, Medical Research Center Oulu, OYS Aistinelinsairauksien keskus, Oulu University Hospital, Oulu, Finland; 3 Department of Oncology and Radiotherapy, Research Unit of Health Sciences and Technology, Medical Research Center, OYS E-talo, Oulu University Hospital, University of Oulu, Oulu, Finland

**Keywords:** Oral cancer, Oral cancer

## Abstract

**Background and purpose:**

Radiation therapy (RT) is a cornerstone of head and neck squamous cell carcinoma (HNSCC) treatment, often used alongside surgical approaches, delivering curative doses between 50–70 Gy. Pre-RT dental screenings aim to prevent oral complications by extracting teeth with poor prognosis, particularly in high-dose areas exceeding 40 Gy. The aim of the present study was to measure the planned radiation doses received on dentition during definitive or postoperative radiation therapy for HNSCC.

**Material and methods:**

This retrospective study analyzed 91 HNSCC cases treated with RT at Oulu University Hospital (2018–2021), assessing radiation doses to dentition, mandible, and parotid glands across different tumor sites.

**Results:**

Results showed that ipsilateral RT spared contralateral and frontal dental regions more effectively than bilateral RT, particularly in oral cavity and oropharyngeal cancers. Conversely, hypopharyngeal and laryngeal cancers rarely exposed dentition to doses above 40 Gy. Bilateral RT often exceeded the 40 Gy threshold in mandibular and parotid regions, particularly in oral cavity cancers, underscoring the need for precise dose planning to balance tumor control with oral health preservation.

**Conclusion:**

The findings highlight that ipsilateral RT can reduce the need for pre-RT dental extractions in contralateral regions and provide a basis for optimizing dental care strategies. By understanding dose distributions, balance can be addressed between minimizing oral health impacts and ensuring effective HNSCC treatment.

## Introduction

Head and neck squamous cell carcinoma (HNSCC) refers to malignant tumors of the lips, oral cavity, various regions of the pharynx, nasal cavity and its sinuses, larynx, and salivary glands. The three main risk factors are tobacco, alcohol, and in oropharyngeal cancer, human papillomavirus (HPV) [1]. According to the Finnish Cancer Registry in 2021, there were 762 new cases of oral and pharyngeal cancer [2].

Radiation therapy (RT) plays significant role in HNSCC treatment, with or without surgical approaches [3]. The doses for curative-intent radiation therapy typically range between 50–70 Gy, with the standard practice being to administer 2 Gy per fraction, five times a week. HNSCC predisposes patients to many dental complications. RT adverse effects include mucositits, trismus, xerostomia, radiation caries and osteoradionecrosis (ORN). Therefore, it is crucially important to include oral health in the treatment to prevent and treat these management of patients irradiated for HNSCC [4]. In addition, pre-RT dental extractions affect the patient’s masticatory system function and additionally their nutrition.

Pre-RT dental screenings aim to identify and eliminate oral infection foci, in order to prevent complications both locally and systemically during and after oncological treatments [5]. In patients with HNSCC the size of the tumor and lymph node status affect radiation doses in the mandible and dentition, with doses exceeding 30 Gy associated with increased risk of osteoradionecrosis in mandible in the context of long lifespan [6]. Teeth that are unlikely to endure a lifetime, ought to be extracted. If the radiation dose maximum in tooth is expected to be above 60 Gy, direct tooth damage occurs [7]. With the radiation dose between 30 and 60 Gy, there is 2–3x increased tooth dose-damage relationship, which is possibly partly due to detrimental impact of radiation on salivary glands.

We used a 40 Gy near-maximum dose (D₂%) threshold to compare the radiation doses delivered to the dentition during RT. Previous studies have shown that microscopic tissue damage can occur with even lower doses, even with 30 Gy [7]. However, the development of ORN typically requires higher radiation doses, around doses of 50–60 Gy in the mandible [8]. It is known that RT complicates dental implant treatment, especially when implant-bed-specific dose exceeds 40 Gy D₂% [9]. When the magnitude of radiation dose in the dentition is better understood, it is possible to plan and implement implant treatment more comprehensively. Even without strong evidence, 40 Gy has commonly regarded as an empirical threshold used in national guidelines concerning radiation doses to the dentition. For our study, the threshold of 40 Gy D₂% was chosen because it is, at our knowledge, most commonly accepted threshold, and widely used in Finland. Although it could also be determined in other ways and the literature shows variability in the appropriate threshold value. In this case, we aimed to assess radiation doses affecting the dentition rather than focusing on those leading to ORN, which is why we selected a relatively low threshold.

Salivary glands, including the parotid and sublingual glands, as well as minor salivary glands, are organs at risk (OAR) during radiotherapy for HNSCC. Non-reversible tissue damage may occur at radiation doses as low as 10 Gy [10]. Minimizing the radiation dose to the salivary glands to the as low as reasonable (ALARA principle) is crucial to preserve their function, as xerostomia increases the risk of radiation caries and ORN. The QUANTEC group has defined the OAR dose limit of <20 Gy when only one parotid gland is spared and <25 Gy when both glands are spared (QUANTEC criteria for parotid glands) [11]. Adhering to these guidelines helps prevent severe xerostomia, characterized by a long-term reduction in saliva production to less than 25 percent of normal levels. Nevertheless, in many cases, it may be necessary to compromise salivary gland protection to effectively treat the cancer, requiring careful consideration of potential side effects in the patient’s oral care.

There is no precise information available regarding on the radiation doses to which various dental areas are exposed during the HNSCC treatment, so decisions about dental extractions are based on the target dose. In this study, we evaluated the planned radiation doses delivered to the dentition, mandible, and parotid glands. In the future, this information can enable to recommendations of more specific dental care for various dental regions during HNSCC treatments in the future.

HNSCC has known to have high incidence of lymph node metastasis. When primary tumor is not approaching the midline, and nodal stage is moderate, ipsilateral RT can in certain cases be sufficient. In this study, we aimed to explore differences in radiation distribution to the dentition between bilaterally and ipsilaterally administered RT across various tumor sites in HNSCC.

## Material and methods

### Population

This study is a retrospective observational cohort study, and it has been reported in accordance with the STROBE (Strengthening the Reporting of Observational Studies in Epidemiology) guidelines [12]. As this study is retrospective review, informed consent was not required, provided that no identifiable data were included. All the patients treated with RT for HNSCC at the Oulu University Hospital’s Department of Oncology and Radiotherapy during the years 2018–2021 were retrospectively assessed for eligibility. The chosen time frame ensures uniform treatment techniques and contouring guidelines for the available patient data. To be eligible for the study population, the patients had to be over 18 years of age receiving curative intent definitive or adjuvant RT. At our institution, all patients who receive RT for HNSCC undergo dental evaluation prior to the start of treatment. The final study cohort comprised 91 patients. The study had a registry research permit granted by the University of Oulu.

Demographic data (Table 1), the patient’s age and sex, the primary tumor location, radiation dose at the primary tumor, TNM classification according to 8^th^ edition, histology, the HPV status of the cancer, the treated neck lymph node regions and how RT were fractioned, were collected manually from electronic health record system (EHR). TNM stage, HPV status, and chemotherapy was collected to describe the patient population, although this information was not included in the analyses because of limited statistical power.

**Table 1:** Demographic data of 91 patients treated with definitive or postoperative RT.

Sex (*n*)	Women	27% (25)
	Men	73% (66)
Age (years)	Median	68,0
	Range	31,0–96,0
Site of primary tumor (n), and planned RT dose in the primary (Gy)	Nasopharynx	6% (5), 50–70
	Nasal cavities	7% (6), 50–70
	Hypopharynx	7% (6), 66–70
	Larynx	9% (8), 70
	Parotid glands	8% (7), 60–70
	Oral cavity	31% (28), 60–70
	Oropharynx	34% (31), 60–70
Stage (*n*)	I	10% (9)
	II	14% (13)
	III	33% (30)
	IVA	38% (35)
	IVB	4% (4)
Histology (*n*)	Squamous cell carcinoma	86% (78)
	Other	14% (13)
Treatment (*n*)	Postoperative	40% (36)
	Postoperative chemo^a^	14% (13)
	Definitive-RT	11% (10)
	Definitive-RT with chemo^a^	35% (32)
Completed treatments	95%	

^a^Cisplatin as radiation sensitizer.

### Treatment and radiation therapy

The treatment was planned according to the Finnish HNSCC guideline and defined by the multidisciplinary tumor board meeting. The final decision on whether the patient should undergo RT was made in collaboration with the oncologist during the patient’s first appointment. HNSCC treatments were planned using consensus guidelines in delineation of the primary tumor [13] and selecting of appropriate lymph node levels [14, 15]. Guidelines were also utilized for delineation of individual lymph node levels [16] and organs at risk [17].

Radiotherapy was given typically in 2 Gy daily fractions to a total dose of 70 Gy in definitive treatment and 60–66 Gy in postoperative treatment. Treatments were given using intensity modulated radiotherapy (IMRT) technique and mainly volumetric modulated arc therapy (VMAT). Simultaneous integrated boost (SIB) technique was used for different dose levels in primary tumor and elective lymph node areas, where the radiation dose was typically 50 Gy in 2 Gy equivalent dose (EQD2). The treatments were carried out following QUANTEC criteria and ALARA principle for the OARs.

### Radiation planning

The planning 3 T MRI (Siemens Healthineers, Forchheim, Germany) and contrast enhancement CT scans (Toshiba Aquilion LB, Canon Medical Systems, Otawara, Japan) were performed in treatment position. The MRI included T1 and T2 sequences, as well as a contrast–enhanced T1 sequence. The RT plans were developed during treatment course using the Varian Eclipse treatment planning software (version 16, Varian Medical Systems, Palo Alto, CA, USA). The plan reveals the radiation dose targeted at the primary tumor and the distribution of doses in OARs.

### Statistical methods

The analyses in this study are primarily descriptive. Summary measures included mean doses and ranges. In addition, percentages of values exceeding threshold doses were analysed by region. Following identification, the doses of PTV, OARs and dental sectors were obtained from dose-volume histograms (DVHs) with the Varian Eclipse. All reported doses to the PTV, OARs and dentition were physical cumulative doses derived from the DVHs. Tabulating was presented using IBM SPSS Statistics (Version 30). The analyses, particularly those involving small subgroups, should be interpreted as descriptive and exploratory. Therefore, no a priori sample size or power calculations were performed.

### Contouring and dose calculations

The contouring of the dentition, mandible, and OARs was performed retrospectively, primarily in a clinical setting, by trained staff familiar with the impact of inter- or intra-observer variability. Each completed treatment plan with contours was reviewed by a hospital physicist specialized in radiotherapy. The reported DVH analyses are secondary analyses of the planning data.

Undelineated sectors and absent glands were excluded from analyses. In some cases, not all sectors could be delineated in patients with oral cavity tumors due the absent part of mandible after surgical treatment, so these sectors were excluded from denominator when calculating percentages. Accordingly, the percentages in these instances refer to a smaller subset of the subgroup. For surgically removed parotid and submandibular glands, the resected glands were excluded from QUANTEC-based calculations, as QUANTEC criteria do not apply to absent glands.

**Fig. 1:**
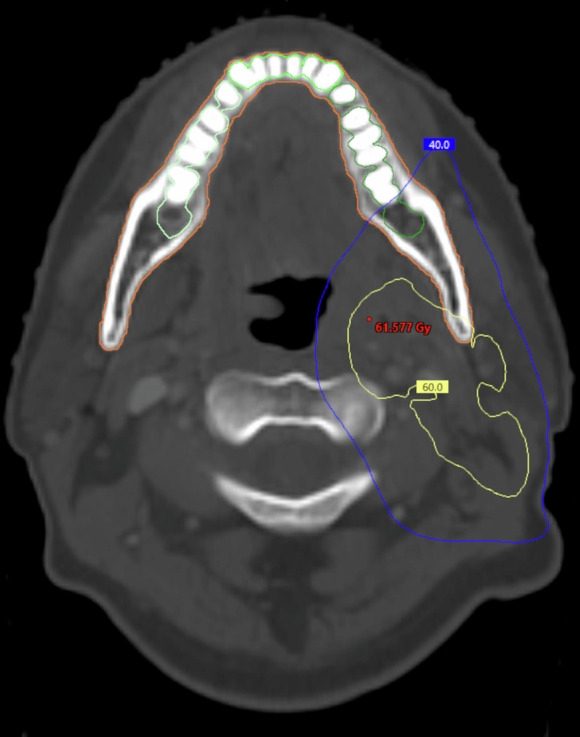
CT scan of patient with oral cavity cancer who received ipsilateral RT with 66 Gy. The figure shows that 40 Gy threshold barely reaches even the molars.

**Fig. 2:**
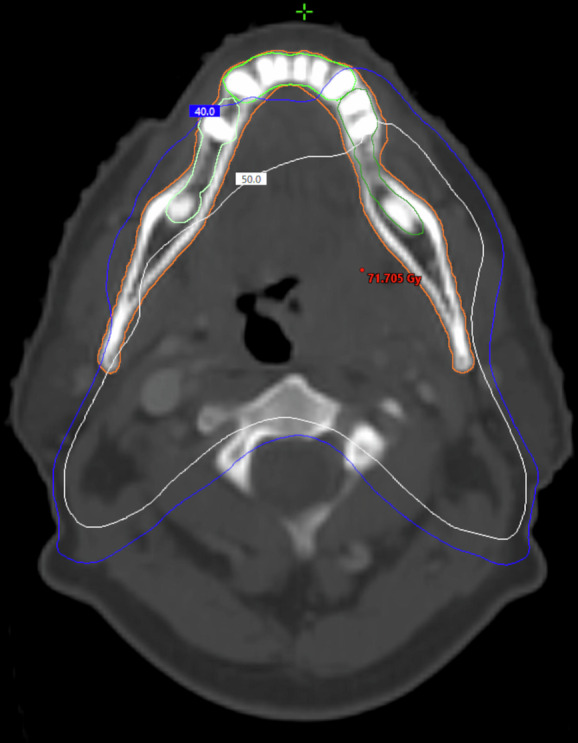
CT scan of patient with oropharyngeal cancer who received bilateral RT with 70 Gy. The figure shows that anterior region is almost completely spared from 40 Gy radiation dose.

Dentition were divided into permanent tooth sextants by using the canines as reference points, and those dental sector’s locations were delineated (Figs. 1 and 2). The frontal sectors included incisors and canines, back sectors included premolars and molars. RT planning CT-scans were used to outline the dental sectors and mandible. From planning CT, the window and viewing level was set to bone density. For each dental sector a new structure was created. Teeth were outlined including their roots up to the enamel, aiming to incorporate the entire tooth into the target delineation.

For patients with missing teeth or parts thereof, sector division was carried out using the jawbones landmarks, extending to the apical level of soft tissue. If some or all teeth were missing from the sector to be contoured, the central mandibular sector was defined using the mandibular symphysis, the midline of the alveolar ridge and canine teeth as anatomical landmarks. The lateral mandibular sectors were defined using the canine teeth, and the anterior border of the mandibular ramus. In addition, any visible residual root canals at sites of extracted teeth were used as supplementary landmarks. When teeth were missing, the maxillary central sector was contoured using the anterior nasal spine and the canine eminence as anatomical landmarks, also residual root canals were used. Correspondingly, the lateral sectors were contoured using the canine eminence and the maxillary tuberosity as anatomical landmarks.

The sectors allowed for the examination of radiation doses to specific dental regions. Parotid and submandibular glands were delineated on soft-tissue CT window settings, using RT planning MRI when necessary for better contrast. After OARs and dental sectors were delineated, the mean dose (Dmean) and the near maximum dose (D₂%) in these locations were exported.

The maximum dose for each dental area of each patient was determined and it was assessed who exceeded 40 Gy D₂% threshold. In bilaterally treated patients we chose to report separately left and right instead of ipsilateral and contralateral neck regions. It was not considered straight forward to define ipsilateral and contralateral neck regions in all cases as some tumors were located in the midline and some patients had metastatic nodes bilaterally.

For parotid glands, it was assessed which patients reached the QUANTEC-criteria, meaning whether both parotid glands achieved doses below 25 Gy or if one parotid gland received dose below 20 Gy.

Patients were divided into two groups based on the targeting of the treatment. The first group (71 patients) comprised patients who received radiation to both sides of the neck lymph node areas. The second group (20 patients) consisted of patients who received treatment only on the ipsilateral neck lymph node areas.

## Results

### Oral cavity cancers

In bilaterally treated oral cavity cancers, the D₂% typically exceeded 40 Gy in the mandibular dentition and, almost as often, in the maxillary dentition (Table 2). In ipsilateral treatments, the 40 Gy threshold was exceeded in the ipsilateral maxillary and mandibular dentition in 75% of patients, and in the maxillary frontal dental area, this occurred in 63% (Table 3). Ipsilateral treatment spared the contralateral premolars and molars, with only 13% of maxillary and 25% of mandibular contralateral dentition receiving doses exceeding 40 Gy (Table 3, Fig. 1). Maxillary frontal dentition typically received doses below 40 Gy.

**Table 2:** Percentage of patients who received RT in various dental areas in bilateral neck.

	Exceeding 40 Gy D₂% Dmean dose range (Gy)					
Primary (*n*)	Maxillary incisors, canines	Maxillary right premolars, molars	Maxillary left premolars, molars	Mandibular incisors, canines	Mandibular right premolars, molars	Mandibular left premolars, molars
Oropharynx (27)	44% 2.7–71.4	67% 4.3–70.3	70% 7.8–71.8	67% 29.2–71.9	78% 23.8–70.2	89% 25.3–73.3
Oral cavity (20)	65% 12.3–68.7	59% 11.3–70.8	76%^a^ 16.2–70.2	90% 28.0–69.2	94%^b^ 11.1–69.7	100% 43.7–68.9
Larynx (8)	0% 1.1–38.7	12.5% 1.8–49.4	0% 1.7–34.3	12.5% 20.0–44.0	25% 20.6–50.5	37.5% 24.9–49.2
Hypopharynx (6)	0% 0.2–31.7	0% 0.3–33.2	0% 0.3–28.1	17% 0.5–45.2	50% 0.8–60.3	33% 0.9–63.8
Nasopharynx (4)	50% 23.0–53.9	50% 27.0–58.9	100% 45.3–56.1	0% 0.5–36.7	50% 0.5–50.1	50% 0.5–42.2
Nasal cavities (6)	100% 50.6–72.9	67% 3.7–67.8	67% 5.9–72.5	0% 0.3–29.1	17% 0.3–42.5	33% 0.2–53.55

^a^Three patients could not be delineated.

^b^Two patients could not be delineated.

**Table 3:** Percentage of radiation dose levels exceeding 40 Gy dose in various dental regions in patients who received RT to the ipsilateral neck.

Primary (*n*)	Exceeding 40 Gy D₂% Dmean dose range (Gy)					
	Maxillary incisors, canines	Maxillary ipsilateral premolars, molars	Maxillary contralateral premolars, molars	Mandibular incisors, canines	Mandibular ipsilateral premolars, molars	Mandibular contralateral premolars, molars
Oropharynx (4)	25% 23.0–43.3	75% 34.6–67.4	75% 23.0–61.7	50% 36.0–65.0	100% 43.3–69.0	50% 24.0–62.2
Oral cavity (8)	38% 16.9–68.0	75% 21.5–66.5	13% 11.1–61.6	63% 25.0–63.7	75% 39.0–67.9	25% 18.9–55.7
Parotid glands (7)	29% 21.0–62.2	71% 33.7–68.7	0% 14.5–35.8	43% 16.8–68.4	86% 29.0–70.3	14% 18.3–43.2
Nasopharynx (1)	100% 56.2	100% 56.8	0% 26.5	0% 37.3	100% 54.5	0% 18.8

### Oropharyngeal cancers

In bilaterally treated oropharyngeal cancers, the dentition was spared to a slightly greater extent than oral cavity cancers treated in the same manner (Table 2, Fig. 2). However, most patients exceeded the D₂% 40 Gy threshold in mandibular dentition, as well as in the premolars and molars of the maxillary dentition. When treated ipsilaterally, the contralateral dentition in oropharyngeal cancers is generally spared to a lesser extent compared to oral cavity cancers (Table 3). Specifically, 75% of patients exceeded 40 Gy in contralateral maxillary premolars and molars, and 50% in contralateral mandibular dentition (Table 3).

### Hypopharyngeal and laryngeal cancers

Hypopharyngeal and laryngeal cancers are typically treated bilaterally, but in these cases, the radiation dose rarely extends to the maxillary dentition (Table 2). In the mandible dentition, the dose typically does not affect the anterior region and is very limited to the mandibular premolars and molars.

### Nasopharyngeal and nasal cavities cancers

Nasopharyngeal and nasal cavity cancers are treated bilaterally (Table 2). Radiation dose exceeds 40 Gy in maxillary dentition, while mandibular anterior regions are always spared.

### Parotid gland cancer

In parotid gland cancers, RT is delivered to the affected side of the neck, sparing contralateral dentition (Table 3). The contralateral Dmean range was between 3.0–17.5 Gy. Additionally, the anterior dentition was spared in both jaws.

### Parotid gland as organ at risk

Among all bilaterally treated patients, 68% met the QUANTEC sparing criteria for parotid glands (Table 4). In bilaterally treated oropharyngeal cancers, 52% met these criteria. For hypopharyngeal cancers 67% and in laryngeal cancers 75% of patients’ parotid glands met the QUANTEC sparing criteria.

**Table 4:** Radiation dose for mandible, parotid gland and submandibular gland in patients who received bilateral neck RT.

Primary (*n*)	Mandible	Parotid gland		The percentage of parotid glands that were spared to the QUANTEC criteria	Submandibular gland	
	Mean dose range (Gy) (Mean) Max dose range (Gy) (Mean)	Mean dose range (Gy)			Mean dose range (Gy)	
		Right	Left		Right	Left
Oropharynx (27)	29.6–69.4 (45.2) 46.7–71.8 (65.8)	13.6–59.0	14.8–65.7	52%	31.7–70.0^c^	34.2–71.6^d^
Oral cavity (20)	26.1–57.0 (46.5) 60.2–70.0 (66.0)	6.0–43.2	7.0–64.0	80%	28.3–68.7^a^	8.7–68.7^b^
Larynx (8)	15.7–49.1 39.8–69.8	3.4–30.9	3.1–37.7	75%	35.3–65.2	35.6–67.6
Hypopharynx (6)	0.8–42.8 2.0–68.3	0.8–29.6	0.8–37.3	67%	7.5–66.0	6.2–69.4
Nasopharynx (4)	3.4–54.1 24.2–69.2	1.2–35.0	0.6–31.6	75%	63.3–68.4	46.9–63.5
Nasal cavities (6)	0.1–51.1 0.3–70.5	0.03–22.0	0.03–46.9	83%	0.06–56.2	0.06–64.3

^a^16 had been surgically removed.

^b^12 had been surgically removed.

^c^4 had been surgically removed.

^d^5 had been surgically removed.

In ipsilateral radiation, parotid glands were below Dmean 25 Gy in 25% in both oral cavity and oropharynx cancers (Table 5).

**Table 5:** Radiation dose levels for mandible, parotid gland and submandibular gland in patients who received RT to the ipsilateral neck.

Primary (n)	Mandible	Parotid glands		Submandibular glands		
	Mean dose range (Gy) (Mean) Maximum dose range (Gy) (Mean)	The ipsilateral percentage of those fell below 25 Gy mean dose	Contralateral mean dose range	Ipsilateral mean dose range	Contralateral mean dose range	
Oropharynx (4)	25.0–53.8 (41.6) 54.4–70.3 (64.0)	25%	7.1–20.4	65.6–68.2^d^		12.7–59.0
Oral cavity (8)	23.2–42.3 (35.3) 39.1–70.0 (60.8)	25%^b^	1.5–19.9	55.7^c^		7.7–20.2
Parotid glands (7)	22.6–48.0 60.3–72.6	0%^a^	3.0–17.5	60.4–70.0		10.3–29.6
Nasopharynx (1)	25.4 55.5	100%	7.1			12.9

^a^6 had been surgically removed.

^b^One has been surgically removed.

^c^7 had been surgically removed.

^d^2 had been surgically removed.

### Mandible RT dose

In bilaterally treated oral cavity and oropharyngeal cancers, the Dmean and Dmax ranges in mandible were narrower compared to other bilaterally treated cancers (Table 3). Specifically, in oral cavity cancers, the mandible received Dmean values ranged between 26.1–57.0 Gy (population mean 46.5 Gy), with Dmax’s mean 66.0 Gy. For oropharyngeal cancers, the mandible’s range of the Dmean was 29.6–69.4 Gy (population mean 45.2 Gy), while the Dmax’s mean was 65.8 Gy. Tumors in the oral cavity and oropharynx, received higher doses of radiation to the mandible compared to other tumor sites. Among patients who received bilateral treatment, 80% of those with oral cavity cancers, 63% of those with oropharyngeal cancers and 75% of those with nasopharyngeal cancers had a mandibular Dmean exceeding 40 Gy. In contrast, at other bilaterally treated tumor sites, the mandibular Dmean exceeded 40 Gy in no more than one-third of patients.

When treating ipsilaterally mandible reached lower radiation doses. In oral cavity cancers, the mandible received the range of the Dmean 23.2–42.3 Gy (population mean 35.3 Gy) and maximum doses Dmean 60.8 Gy. For oropharyngeal cancers, the mandible’s range of the Dmean was 25.0–53.8 Gy (population mean 41.6 Gy), and maximum doses Dmean was 64.0 Gy. In ipsilateral treatments, 50% of oropharyngeal patients had a mandibular Dmean exceeding 40 Gy, whereas in other ipsilateral treated tumor sites the mandibular Dmean exceeded 40 Gy in either less than one-third of patients or none.

## Discussion and conclusion

Specialized dental care is crucial in preventing and managing oral complications associated with RT for HNSCC. Indications for dental extractions are to reduce infection foci, radiation caries and developing ORN after RT. Communication and effective collaboration between radiation oncologists and dental specialists are essential for optimizing patient care and preventing post-treatment oral complications [18]. According to the literature, the incidence of ORN in HNSCC varies widely, from 2% to 23% [19–22]. Modern techniques like 3D conformal therapy and IMRT appear to have reduced the incidence to 6% or less [19]. It has been found that the incidence of ORN is higher in patients who have underwent dental extractions before RT compared to those who did not require dental extractions [23]. Anyhow, it is considered safer to do extractions before the RT than afterwards.

Even though modern radiation techniques are reducing the ORN risk, it remains a relevant complication for HNSCC patients undergoing RT. After RT, predisposing risk factors for ORN include infectious foci in the teeth, poor oral hygiene, dental extractions after RT, pre-RT operative treatment of the mandible, and oral cavity or oropharyngeal tumor sites.

The incidence of ORN varies in the literature and there are several limitations in previous literature, such as small sample sizes, retrospective small cohorts, variability in the dental management protocols and appliances, and the timing of dental interventions, which leads to inconsistent results [24–26]. Specific data on the incidence of ORN in HPV-positive HNSCC patients is very limited in literature, yet one study highlighted that the incidence of ORN did not notably decrease by the introduction of IMRT, particularly in HPV-positive oropharyngeal cancer patients, suggesting a persistent risk in this group [27]. On the other hand, HPV patients are more often smoke free and healthier, which may enable maintaining better oral hygiene and furthermore, have positive impact in the incidence of ORN. In a study that investigated patient and treatment-related risk factors for ORN in head and neck cancer (HNC) patients, 74% who developed ORN after RT had received a radiation dose of 66 Gy or higher [21].

Radiation is thought to lead into hypoperfusion and hypovascularity, impairing bone health and healing, particularly affecting periosteal blood flow [28]. ORN is known to be predominant in the mandible, which is because of the poor vascular supply and higher bone density compared to maxilla, so more aggressive pre-RT extractions are preserved for mandibular dentition [29].

Dentition are rehabilitated prior to RT thoroughly, and this may be the reason why cases of ORN are rarely seen in our clinic. As aggressive dental extractions have also a negative impact on patient, we considered to examine whether dental extractions could be performed less extensively. Because ORN cases are very rare, and none of the patients in our cohort developed ORN during the data collection period, we were unable to compare the results with the incidence of ORN. In patients with oropharyngeal and nasopharyngeal cancer, challenges in dental rehabilitation using implant therapy may already be observed at RT doses exceeding 30 Gy [30]. Microvascular damage has been reported even at this lower threshold than previously presented. Setting a threshold like 40 Gy is a compromise well supported by existing literature, reflecting that vascular damage and impaired bone healing capacity begin to become clinically notable above this dose level [31]

In this study, we focused on the planned radiation doses, and the distribution of the doses delivered to the dentition. No statistical testing was performed, and results are reported qualitatively due to ongoing but at the moment insufficient efforts to select relevant endpoints and access patients’ records of clinical adverse effects. An impact on the dentition and parotid doses and the occurrence of ORN depends on whether the treatment for the HNSCC patients is ipsilateral or bilateral. Our clinical experience is that surgeons more often choose ipsilateral treatment, while oncologists prefer bilateral treatments, which is also recognized in the literature [4]. This has also considered in recent Finnish guideline, which recommends more ipsilateral radiation in selected cases. Optimal choosing of the appropriate target area and keeping the treatment volume as limited as feasible is the most sufficient way to diminish unnecessary radiation dose outside the treatment area.

Ipsilateral RT was found to spare more dentition in patients with oral cavity cancers, especially maxillary frontal teeth and contralateral premolars and molars. More precise targeting in oral cavity and oropharyngeal cancers may reduce the need for pre-RT extractions. With current techniques, radiation doses over 40 Gy can often be avoided in contralateral dentition and anterior teeth, particularly in oropharyngeal, nasopharyngeal, and hypopharyngeal cancers. In hypopharyngeal and laryngeal cancers, dentition doses typically remain below this threshold due to tumor location, supporting a more conservative extraction approach.

One study that focused only on oropharyngeal cancers [6] had similar results: ipsilateral premolars and molars received higher radiation dose than contralateral premolars and molars. Our study design differs, as we examined the distribution of radiation to the dentition by sectors. Still, it is in line with the above-mentioned study, where it was noticed that the number of metastatic nodes increases dental doses, as radiation target volumes increases. Our study aligns with a similar study regarding the distribution of radiation doses, which addressed the necessity of tooth extractions in HNC [32]. According to our results tumor site and the targeting of RT, either bilaterally or ipsilaterally, has high association to the distribute of the radiation dose in the dentition.

Dental care practices for HNSCC patients undergoing RT vary due to the complexity of managing the oral health complications associated with RT. Pre-RT assessment includes assessing dental caries, periodontal disease, and the need for extractions. The necessity and extent of pre-RT dental extractions is also debated topic. In our study, dental extractions were performed before of the RT. There is conflicting information in the literature regarding the timing of dental extractions in relation to RT, but recent studies support performing pre-RT dental extractions [33]. Ideally there should be at least 7-14 days between dental extractions or other surgical treatments before to RT [34]. However, general consensus is that the extent of dental extractions requires balancing, as post-extraction’s caused surgical trauma can lead to ORN, and at the same time it is necessary to avoid delays in the initiation of RT to prevent severe prognostic disadvantages. Almost all treatment guidelines recommend extracting all moderate to severely periodontally involved teeth, when they are expected to receive a high dose of RT [35]. The importance of knowing the doses is particularly emphasized in patients who have undergone extensive dental extractions prior to RT. In a study examining patients with oropharyngeal cancer [36], it was found that the functional dentition of HNC patients is poorer than that of the general population. Furthermore, comparing patients’ dental status to the tumor sub-site revealed statistical differences. The study seems to be in line with our view that, when planning patient treatment, greater consideration should be given to the tumor location as well as the individual needs of the patient. This should be done while balancing the preservation of masticatory function against the risk of ORN, particularly when prolonged survival is anticipated. In addition, patients experience the pre-RT dental extractions as burdensome and detrimental to their quality of life [37]. By understanding dental doses more comprehensively, it may be possible to preserve more dentition prior to RT. Also, it is necessary to consider what would be the optimal amount of dental care without causing a delay in RT.

AI-based auto-segmentation and adaptive radiotherapy (ART) are promising approaches to enhance the precision of RT for HNSCC. These advancements may particularly reduce exposure to critical structures like dental tissues and parotid glands. AI-models have demonstrated higher accurancy in segmenting OARs compared to traditional atlas-based methods, indicating more precise and consistent segmentation in the future [38, 39]. Nevertheless, further development is required to enhance the performance and generalizability of these methods for auto-segmentation.

The strength of our study includes clear and reproducible methods, tumors and OARs were delineated by experienced clinician in close collaboration with multidisciplinary team. Delineation was complied with consensus guidelines. In addition, the latest validated analysis methods (IMRT, VMAT) were used to calculate radiation doses. The group of subjects was representative in terms of age and gender, and patients of all HNSCC tumor sites were included in the study.

The sample size (91 patients) is relatively small, which may limit the statistical power and generalizability of some findings. Although dividing dentition into sectors allows for a more detailed analysis, this approach may overlook differences in radiation exposure between individual teeth. Standardizing the method can be challenging, but on the other hand, the method considers patients with missing teeth or an atypical dental map. As potential confounding factor, it should be noted that baseline dental status and the presence of periodontal disease were not assessed in this study. In addition, the extent of dental extractions performed prior to RT was not accounted for. These factors are likely to influence both dental outcomes and the risk of ORN, which are important to consider when interpreting the implications of the study for dental management.

## Permissions

Permission to use the registry data was granted by the Northern Ostrobothnia Hospital District (approval number: 176/2020).
